# Effects of Chinese medicine for COVID-19 rehabilitation: a multicenter observational study

**DOI:** 10.1186/s13020-022-00654-z

**Published:** 2022-08-22

**Authors:** Linda Li-Dan Zhong, Yi-Ping Wong, Chor-Yin Leung, Bo Peng, Zhi-Xiu Lin, Vivian Chi-Woon Wong Taam, Yi Luo, Hai-Yong Chen, Chao-Dong Chao, Chor-Fung Wong, Freddie Shung-Chi Tam, Kui Chan, Kwan-Yiu Lee, Lai-Fun Ho, Alan Yat-Lun Wong, Chi-Fung Choy, Bacon Fung-Leung Ng, Rowena How-Wan Wong, Yi-Bin Feng, Ching Liong, Zhao-Xiang Bian, COVID-19 CM Research Working Group

**Affiliations:** 1 School of Chinese Medicine, Hong Kong Baptist University, Hong Kong, China; 2 School of Chinese Medicine, The Chinese University of Hong Kong, Hong Kong, China; 3 School of Chinese Medicine, LKS Faculty of Medicine, The University of Hong Kong, Hong Kong, China; 4 United Christian Nethersole Community Health Service - The Chinese University of Hong Kong Chinese Medicine Clinic Cum Training and Research Centre (Tai Po District), Hong Kong, China; 5 HKFTU Workers’ Medical Clinics - Hong Kong Baptist University Chinese Medicine Clinic Cum Training and Research Centre (North District), Hong Kong, China; 6 Pok Oi Hospital - Hong Kong Baptist University Chinese Medicine Clinic Cum Training and Research Centre (Kowloon City District), Hong Kong, China; 7 The Hong Kong Tuberculosis Association – The University of Hong Kong Chinese Medicine Clinic Cum Training and Research Centre (Southern District), Hong Kong, China; 8 Pok Oi Hospital - The Chinese University of Hong Kong Chinese Medicine Clinic Cum Training and Research Centre (Yuen Long District), Hong Kong, China; 9 Pok Oi Hospital - The Chinese University of Hong Kong Chinese Medicine Clinic Cum Training and Research Centre (Shatin District), Hong Kong, China; 10 Haven of Hope - The Chinese University of Hong Kong Chinese Medicine Clinic Cum Training and Research Centre (Sai Kung District), Hong Kong, China; 11 Department of Medicine, Tseung Kwan O Hospital, Hospital Authority, Hong Kong, China; 12 Chinese Medicine Department, Hospital Authority, Hong Kong, China

**Keywords:** Covid-19, Rehabilitation, Observational study, Chinese Medicine, Respiratory system, Lung function, Quality of Life, Body constitutions

## Abstract

**Objectives:**

This study aimed to evaluate the effects of Chinese Medicine (CM) on the health condition of the post-COVID-19 patients, particularly with the CM Syndrome diagnosis and Body Constitutions (BC), as well as related clinical characteristics.

**Methods:**

150 participants who had COVID-19 and discharged from Hong Kong public hospitals were recruited. They were provided with three to six months of CM treatments, during which assessments were made per month and at follow-up on their CM syndromes, BC, lung functions, and other medical conditions. This study was divided into two parts: (1) Retrospective survey: medical history of participants during COVID-19 hospitalization was collected during the baseline visit; (2) Prospective observation and assessments: clinical symptoms, lung functions, and BC status were evaluated in participants receiving CM treatment based on syndrome differentiation and clinical symptoms.

**Results:**

The median hospitalization period was 16 days. Symptoms were presented in 145 (96.6%) patients at the day they were diagnosed with COVID-19. Fever, fatigue, and dry cough were the most common symptoms, exhibiting in 59.3% (89 of 150), 55.3% (83 of 150), and 46% (70 of 150) participants, respectively. Among the 150 post-COVID patients, majority (71.3%) were of the two particular post-COVID CM Syndromes (Qi Deficiency of Lung and Spleen, and Qi and Yin Deficiency). Upon CM treatment, there was an observable increase in participants reaching a balanced BC (i.e. healthy body conditions). The increase was observed to be more prominent in those without the particular CM Syndromes compared to those with the CM Syndromes. Main clinical symptoms in participants with the CM Syndromes decreased upon CM treatment. Occurrence of fatigue also dropped after CM treatment though not all accompanied clinical symptoms were resolved fully. Further to the improvement in terms of CM assessments, lung functions of the participants were found to show improvement after treatment. Both the performance in 6MWT and scores in the LFQ improved upon CM treatments (*P* < 0.05).

**Conclusion:**

This study provided evidence for individualized CM treatment on COVID-19 rehabilitation concerning the clinical symptoms improvements, lung functions improvement, and achieving a balanced BC. It is believed that CM may be a key to further promote rehabilitation and resolution of residual symptoms. Long-term large scale follow-up studies on sub-categorising post-COVID patients according to different CM syndromes would be required to further elucidate treatment of persistent symptoms that may be associated with long-COVID.

**Supplementary Information:**

The online version contains supplementary material available at 10.1186/s13020-022-00654-z.

## Introduction

In March 2020, World Health Organization (WHO) declared the outbreak of a coronavirus disease 2019 (COVID-19) to be a pandemic [1]. According to WHO, most patients had mild or uncomplicated forms of COVID-19, while approximately 14% were estimated to be associated with a severe acute respiratory infection and required hospitalization and oxygen support, and 5% required admission to the intensive care unit [2].

With the clearance of SARS-CoV2 infection and recovery from acute clinical symptoms after treatment, the functionality of patients was still impaired. Impairments, whether severely or mildly affected, included not only pulmonary problems but also physical weakness and problems of the psychosocial domains [3]. Even being discharged from hospitals, patients still present significant clinical symptoms such as cough, fatigue, poor appetite, shortness of breath, and poor sleep. Since the sequelae and long-term effects of COVID-19 has not been understood completely, its effects on patient rehabilitation required further study. In Hong Kong (HK), valuable experience was gained on combating the outbreak of a severe acute respiratory syndrome (SARS) epidemic in 2003. A review of the publications during and after the SARS crisis enabled us to get an objective view of the value of adjuvant therapy using Chinese medicine (CM) [4].

China developed the National COVID-19 Diagnosis and Treatment Guidelines, and has constantly updated information about the disease. To facilitate the implementation of integrative Chinese-Western Medicine in COVID-19 management, CM has been recommended in the 7th edition of the national guidelines released in March 2020 [5]. For this reason, CM rehabilitation treatment guidelines were also released simultaneously [6–10]. In order to strengthen the rehabilitation and health management of the hospital discharged COVID-19 patients, CM has been used as a treatment in China during the outbreak. Some of the early papers reported that over 85% of COVID-19 infected patients in China were receiving CM treatment, with an overall effective rate of ≥ 90% [11]. Among these patients, a majority of patients (≥ 60%) showed marked improvement in their symptoms, while 30% of the patients demonstrated a stabilized illness [12].

Although the number of published papers on COVID-19 has increased, many questions still remain and available treatment options are limited [5], in particular regarding the “long COVID” situation [13]. According to the National COVID-19 Diagnosis and Treatment Guidelines, COVID-19 patients suffer from two particular CM Syndromes during their rehabilitation phase—Qi Deficiency of Lung and Spleen Syndrome (“fèi pí liǎng xū”, “肺脾兩虛”) and the Qi and Yin Deficiency Syndrome (“qì yīn liǎng xū”, “氣陰兩虛”). In view to the growing number of hospital-discharged COVID-19 patients in Hong Kong, it is important for CM Practitioners (CMPs) in HK to draw on their experience and summarize the evidence for CM effectiveness on post-COVID-19 rehabilitation. Since early January 2020 when HK first responded to the COVID-19 pandemic, as of 9 Dec 2021, a total of 12,472 COVID-19 cases had been confirmed, and 12,404 patients had been discharged from hospitals, according to the Centre for Health Protection of the Department of Health in HK [14]. This study aimed to collect observational data on the effect of CM on the rehabilitation of the post-COVID-19 subjects, as well as to explore the application of CM Syndromes in differentiating sub-groups of post-COVID-19 subjects to facilitate better health management. The study was done from September 7, 2020 to 30 November 2021.

## Materials and methods

### Overview of study design

This multicenter observational study targeted a recruitment of 150 participants who had been hospitalized in HK public hospitals due to COVID-19 and subsequently discharged upon recovery as well as tested negative of SAR-CoV2. The study consisted of two parts: retrospective analysis of clinical symptoms and CM diagnosis, and prospective analysis of CM Syndrome diagnosis and therapeutic assessments. In the retrospective part, medical history of participants during COVID-19 hospitalization was collected during the baseline visit (“V1”), after obtaining informed consent from the participants. This was done with the aim to delineate participants’ previous disease severity, which included the hospitalization period and comorbidities in the case report by semi-structured questionnaire. In the prospective part, participants received three to six months (“V2” to “V7”) of individualized CM treatment based on CM guidelines on COVID-19 rehabilitation, and individual clinical symptoms. All participants were assessed by questionnaires and lung function tests each month during the treatment period and on a 3-month no treatment follow-up visit (“V8”). The improvement of clinical symptoms and the status of CM Body Constitutions (BCs) were evaluated. The examinations included assessments on CM Syndrome pattern and clinical characteristics, lung functions, and quality of life at each visit for nine months.

### Recruitment

Participants who were discharged from HK hospitals and sought CM consultations at the Chinese Medicine Clinic cum Training and Research Centres (CMCTRs), were recruited. CMCTRs are government-subsidized tri-partite run CM out-patient clinics. They are located in different geographical districts in HK and provided CM services to the general population (Additional file 1: Appendix S1). Participants were enrolled upon their capacity to give written informed consent voluntarily, as well as upon fulfilling the inclusion criteria and without any exclusion criteria. To maximize participants’ compliance to the study, a thorough consent process was provided to all participants, with details of the study schedule, participants’ responsibilities, and support from the study team. Participants were informed of their freedom to withdraw from the study at their discretion without penalty. For dropped out cases, they were contacted by the study team in attempt to document their reasons for withdrawal from the study.

### Eligibility criteria

Inclusion Criteria: We recruited participants (aged 18 years or older) who had been previously diagnosed with COVID-19 and subsequently discharged from local hospitals after treatment, and with negative SARS-CoV2 infection.

Exclusion Criteria: Participants were excluded if they had one or more of the following: (1) inability to communicate (e.g., cognitive impairment), and (2) history of CM allergies.

Termination criteria: Study would be terminated in participants who had withdrawn their consents or participated in another CM interventional research project or exhibited life-threatening situations.

### Interventions

The participants received three to six months of CM treatment based on the recommended prescriptions in the CM clinical practice guidelines for COVID-19 patients, individual CM syndromes, and clinical symptoms [15–18]. Each participant was assessed monthly during the treatment period and at follow-up. The treatment and assessments were conducted by registered CMPs with at least three years of clinical experience. CM prescriptions for the two particular CM Syndromes identified in subjects of post-COVID-19 recovery (Qi Deficiency of Lung and Spleen; Qi and Yin Deficiency) were recommended following CM principles (Additional file 1: Table S1).

### Outcomes measures

Each participant was assessed by questionnaires and lung function tests monthly during the treatment period and on follow-up visit (Additional file 1: Table S2).

Primary outcome of assessment focused on the change in CM diagnostic pattern and clinical characteristics. A scoring checklist was used for assessing the two main CM Syndromes (Qi Deficiency of Lung and Spleen; Qi and Yin Deficiency). The checklist included three main symptoms and five accompanying symptoms. Symptom severity was graded upon a four-point scale (0, 2, 4, 6 and 0, 1, 2, 3), with higher scores indicating higher severity (Additional file 1: Appendix S2, in Chinese only) [19–22]. In addition to CM Syndromes, BC was assessed using nine specific types of questionnaires that were developed based on Traditional Chinese Medicine (TCM) theories. The questionnaires used in this study had been recognized by the China Association of Chinese Medicine as the standard of body constitution [23–25].

Apart from CM perspectives, lung function-related assessments included performance of the 6-min Walk Test (6MWT), risk of chronic obstructive pulmonary disease assessment by the Lung Function Questionnaire (LFQ) [26], assessment on quality of life using the WHO Quality of Life Brief Assessment (WHOQOL-BREF HK validated version) [27], and the frequency of clinic or hospital visits for non-CM treatments during the study period. If any participants exhibited a SpO_2_ of below 95% before or after the 6MWT, they would be required to undergo assessment by handheld basic spirometry tests. In this study, only one participant was required for spirometry assessment due to measurements of SpO_2_ less than 95%. This participant had a 69-day hospitalization period during which mechanical ventilation was required.

### Data collection, management, and analysis

Statistical analyses were performed using GraphPad Prism version 9.3.1 [28] for paired t-test comparisons, and linear mixed modeling in R Studio (version 4.1.2, 2021.09.1 Build 372) [29] to account for missing data and confounding variables. Numerical variables were first tested for normality and were then reported as mean if the data were satisfied with normal distribution; otherwise, the median was used. Comparisons of numerical variables before and after intervention were analyzed by repeated measures of ANOVA. For categorical variables, and chi-squared test or Fisher’s exact test were used for analysis. A P-value of < 0.05 was considered statistically significant.

Linear mixed models were produced in R Studio, using packages: tidyverse [30], lme4 [31], lmerTest [32], ggplot2 [33], readxl [34], dplyr [35], DHARMa [36], and plyr [37]. The models used in analysis below were computed using restricted maximum likelihood estimation (REML), with p-values for fixed effects obtained from Type III Analysis of Variance Table with Satterthwaite’s method of approximation.

“Model 1” represented a linear mixed effects analysis of the relationship between 6MWT performance (i.e. distance achieved in 6MWT) and effect of CM treatment (i.e. time points of CM visits). As fixed effects, we entered time points of CM visits, age, CM symptoms, prior CM consultations (without interaction term) into the model. As random effects, we had intercepts for subjects.

Model 1 = lmer (6MWT Distance ~ Time Points + Age + CM Symptoms + Prior CM Consultations + (1|Subjects), data).

“Model 2” represented a linear mixed effects analysis of the relationship between LFQ scores and effect of CM treatment. As fixed effects, we entered time points of CM visits and we had intercepts for subjects as random effects.

Model 2 = lmer (LFQ Scores ~ Time Points + CM Symptoms + (1|subjects), data).

Visual inspection of residual and Q-Q plots of the above mentioned models did not reveal any obvious deviations from homoscedasticity or normality.

## Results

### Demographics of participants

From 7 Sep 2020 to 30 Nov 2021, 150 discharged COVID-19 patients were recruited and 141 patients had completed the study. Their baseline characteristics were shown in Table 1. Participants were mostly found to be middle-aged [median age: 54 (42–61)], with the youngest at 18 and the oldest at 81. Fourteen participants received mechanical ventilation during hospitalization prior to study, among which 11 were obese or overweight (78.57%); compared to the 136 participants without the need for mechanical ventilation during hospitalization, among which 73 were obese or overweight (53.68%). Twenty-four participants were smokers (16%, 24 out of 150) in this study. The most common medication received during hospitalization was anti-viral drugs (28.67%) while several participants had received corticosteroids (2.67%) and anti-inflammatory drugs (2%). Prior to joining study, all participants had received not more than ten CM consultations in CMCTRs.

**Table 1:** Baseline characteristics of study participants

	Demographic data
No. of participants, N (% of total)	150 (100%)
Female, N (% of total)	96 (64%)
Age, years	54 (42–61)
Height, cm	162 (157–168)
Weight, kg	64 (55–74)
BMI score	23.5 (21.7–27.0)
No. of smokers, N (% of total)	24 (16%)
No. of participants with medical history, N (% of total)	91 (60.7%)
No. of participants requiring mechanical ventilation during hospitalization, N (% of total)	14 (9.3%)
Hospitalization Days, days	16 (12–24)
Treatment received during hospitalization, N (% of total)	
Antiviral drug Interferon treatment Antibody product Antibody cocktail treatment Oxygen support Corticosteroids Anti-inflammatory drugs Immunomodulatory drugs Anti-leprosy drug Others	43 (28.67%) 33 (22%) 23 (15.33%) 15 (10%) 13 (8.67%) 4 (2.67%) 3 (2%) 2 (1.33%) 1 (0.67%) 9 (6%)
No. of participants who received Chinese medicine consultations prior to study, N (% of total)	150 (100%)
1 consultation 2 consultations 3 consultations 4 consultations 5 consultations 6 consultations 7 consultations 8 consultations 9 consultations 10 consultations	19 (12.67%) 24 (16%) 27 (18%) 18 (12%) 12 (8%) 10 (6.67%) 6 (4%) 2 (1.33%) 4 (2.67%) 28 (18.67%)

^#^Unless otherwise stated, the data presented is median with interquartile range [median (IQR)]

### Clinical symptoms, diagnosis and health conditions during hospitalization

Surveys were conducted to retrospectively collect information on the participants’ health conditions during their hospitalization (Table 2). Symptoms were presented in 145 (96.6%) patients at the day they were diagnosed with COVID-19. Fever, fatigue, and dry cough were the most common symptoms, exhibiting in 59.3% (89 of 150), 55.3% (83 of 150), and 46.67% (70 of 150) participants, respectively. Among the 150 participants, 91 (60.7%) had comorbidities before COVID-19 infection. The top three chronic conditions observed among the participants were hypertension (24%), high cholesterol (14. 7%), and diabetes mellitus (12.7%).

**Table 2:** Results from the retrospective survey on participants’ clinical symptoms and chronic illness history

Clinical symptoms	No. of occurrence	% among participants
Fever	89	59.33
Fatigue / Tiredness	83	55.33
Dry cough	70	46.67
Muscle / Joint pain	49	32.67
Headache	42	28.00
Coughing up phlegm	40	26.67
Sore throat	39	25.26
Loss of taste	36	24.00
Loss of smell	36	24.00
Shortness of breath	35	23.33
Diarrhoea	33	22.00
Aversion to cold	31	20.67
Poor appetite	27	18.00
Runny nose	26	17.33
Stuffy nose	21	14.00
Night sweats	18	12.00
Soreness and weakness of waist and knees	15	10.00
Spontaneous sweating	14	9.33
Feverish palms and soles	12	8.00
Nausea/Vomit	6	4.00
Rash	6	4.00
Tinnitus	5	3.33
Coughed up blood	1	0.67
Conjunctival hyperemia	1	0.67

Chronic illness	No. of occurrence	% among participants
Hypertension	36	24.00
High Cholesterol	22	14.67
Diabetes mellitus	19	12.67
Rhinitis	15	10.00
Heart Disease	9	6.00
HBV	9	6.00
Gallbladder Disease	8	5.33
History of Cancer	7	4.67
Eczema	7	4.67
Asthma	6	4.00
Immunodeficiency	5	3.33
Fatty Liver Disease	5	3.33
Depression	5	3.33
Thyroid Disease	5	3.33
Others	12	8.00
Sleep apnea	3	2.00
Gastritis	2	1.3
Thalassemia	1	0.6
Gout	1	0.6
Benignant goiter	1	0.6
History of parotidectomy	1	0.6
History of rhinitis surgery	1	0.6
Recovered from tuberculosis	1	0.6
Anaemia	1	0.6
Uterine fibroids	1	0.6
Pulmonary fibrosis	1	0.6
Hirschsprung’s disease	1	0.6

### CM assessments

Among the 150 participants, 107 (71.3%) participants showed CM syndromes of Qi Deficiency of Lung and Spleen Syndrome and/or Qi and Yin Deficiency Syndrome. 65 participants (43.4%) exhibited both of the particular CM Syndromes, while 27 (18%) were of Qi Deficiency of Lung and Spleen syndrome only and 15 (10%) of Qi and Yin Deficiency only. The remaining 43 (28.7%) participants did not exhibit the particular two CM Syndromes (Table 3A). Though these participants did not exhibit the two particular CM Syndromes, their baseline conditions were mostly found to be similar to those who exhibited the CM Syndromes (Table 3A). Participants without the two particular CM Syndromes were found to be significantly heavier than those with the two Syndromes (weight: p = 0.040; BMI score: p = 0.005). However, both groups were overweight according to the BMI range for the HK population. A significant difference was also detected in the baseline LFQ scores between the two groups. The number of participants who were of the two CM Syndromes decreased upon CM treatment (Table 3B). Comparing between the main clinical symptoms in Qi Deficiency of Lung and Spleen Syndrome and Qi and Yin Deficiency Syndrome, it was observed that their occurrences were similar (Table 4A). The occurrence of accompanied clinical symptom of fatigue was observed to be also similar between the two groups of participants (Table 4B).

**Table 3:** Comparison between participants with or without the two particular CM Syndromes identified in patients in post-COVID-19 recovery period

(A) Baseline characteristics of participants			
	Participants with particular CM syndromes	Participants without particular CM syndromes	P-value
No. of participants, N (% of total)	107 (71.3%)	43 (28.7%)	
Female, N (% of group)	68 (63%)	28 (66%)	1.000
Age, years	54 (43–61.5)	54 (38–60.5)	0.269
Height, cm	162.5 (158–167.2)	160 (156.8–170)	0.591
Weight, kg	63.75 (55–71.4)	70 (60.23–78.25)	0.040
No. of participants requiring mechanical ventilation during hospitalization, N (% of total)	11 (10.19%)	3 (7.14%)	0.750
Hospitalization Days, days	16 (12–18.34)	17 (12–27.5)	0.185
BMI score	23.2 (21.15–23.93)	24.8 (23.05–28.93)	0.005
No. of participants with medical history, N (% of total)	69 (63.89%)	22 (52.38%)	0.185
No. of smokers, N (% of total)	18 (16.67%)	6 (14.29%)	0.852

(B) Changes in the number (and percentage) of participants with the two particular CM syndromes in COVID-19 rehabilitation									
Participants exhibiting:	Qi Deficiency of Lung and Spleen only		Qi and Yin Deficiency only		Both CM Syndromes		Without CM Syndromes		Total N
	N	%	N	%	N	%	N	%	
**V1**	27	18	15	10	65	43.4	43	28.67	150
**V2**	22	14.77	14	9.4	63	42.28	50	33.56	149
**V3**	22	15.17	7	4.83	57	39.31	59	40.69	145
**V4**	21	14.58	14	9.72	51	35.42	58	40.28	144
**V5**	17	11.97	15	10.56	49	34.51	61	42.96	142
**V6**	16	11.35	13	9.22	43	30.50	69	48.94	141
**V7**	18	12.77	9	6.38	40	28.37	74	52.48	141
**V8**	17	12.06	13	9.22	22	15.60	89	63.12	141

^#^Unless otherwise stated, the data presented is median with interquartile range [median (IQR)]

**Table 4:** Occurrence of clinical symptoms

(A) Main symptoms (主症)				
	N	Cough	Coughing up phlegm	Shortness of breath
Qi Deficiency of Lung and Spleen	92	43 (46.74%)	32 (34.78%)	42 (45.65%)
Qi and Yin Deficiency	80	34 (42.5%)	29 (36.25%)	36 (45%)

(B) Accompanied symptoms (兼症)						
	N	Fatigue	Poor appetite	Spontaneous sweating	Post-meal fullness	Loose stool
Qi Deficiency of Lung and Spleen	92	70 (76.09%)	27 (29.35%)	54 (58.70%)	43 (46.74%)	32 (34.78%)

	N	Fatigue	Soreness and weakness of waist and knees	Feverish palms and soles	Tinnitus	Night sweats
Qi and Yin Deficiency	80	58 (72.5%)	49 (61.25%)	25 (31.25%)	23 (28.75%)	32 (40%)

Baseline occurrence (N, %) of clinical symptoms in participants diagnosed with one or both of the two particular CM Syndromes. Both CM Syndromes consist of the main symptoms of cough, coughing up phlegm, and shortness of breath. They also both exhibit fatigue as an accompanied symptom. For the Qi Deficiency of Lung and Spleen CM Syndrome, accompanied symptoms were mostly gastrointestinal symptoms while for Qi and Yin Deficiency CM Syndrome, symptoms include soreness and tinnitus.

In addition to CM Syndrome assessments, BC of participants was also examined. Participants may exhibit a single or a mixture of BCs depending on their health conditions. An overall BC was derived for participants with mixed BCs. As shown in Table 5A, among the different types of BCs, the Qi-deficiency constitution (23.94%) and the Yang-deficiency constitution (19.72%) were found to be most frequently occurring among the post-COVID participants. The constitution of Gentleness represented a balanced constitution, indicating a healthy status of the participant. Such a constitution was found to show 8.45% occurrence among the participants. Apart from Gentleness constitution, all other constitutions represented an unhealthy body condition. Marginal Gentleness constitution was observed for some participants (11.27%) as they were neither in an unhealthy condition nor achieving a balanced constitution yet. Upon CM treatment, it was found that the constitution of Qi-deficiency decreased by the first 3-month treatment from 23.94 to 13.18%, while that of Yang-deficiency only showed observable decrease from 19.7 to 11.9% upon 6-month treatment (Table 5A).

**Table 5:** Distribution of BC types among participants at baseline and after CM treatment. (A) The percentage (and occurrence, N) of each BC type was profiled at baseline and found the two most common BC types were Qi-deficiency and Yang-deficiency which decreased upon treatment at V4 and V7. (B) BC achieving Gentleness increased after CM treatment

(A)								
No. of participants with their BC assessed	142		129		109		111	
BC Types	Baseline (V1) % N		After CM treatment (V4) % N		After CM treatment (V7) % N		Follow-up (V8) % N	
Qi-deficiency (氣虛質)	23.94	34	13.18	17	14.68	16	14.41	16
Yang-deficiency (陽虛質)	19.72	28	19.38	25	11.93	13	14.41	16
Phlegm-wetness /Phlegm-dampness (痰濕質)	10.56	15	9.30	12	11.93	13	13.51	15
Marginal Gentleness / Marginal balance (基本是平和質)	11.27	16	8.53	11	11.93	13	12.61	14
Ying-deficiency / Yin-deficiency (陰虛質)	9.15	13	9.30	12	10.09	11	9.01	10
Gentleness / Balance (平和質)	8.45	12	14.73	19	15.60	17	12.61	14
Wetness-heat / Damp-heat (濕熱質)	6.34	8	3.88	5	4.59	5	3.60	4
Special diathesis / Inherited special constitution (特稟質)	4.93	7	10.08	13	7.34	8	5.41	6
Qi-depression / Qi-stagnation (氣鬱質)	5.63	8	9.30	12	11.93	13	9.91	11
Blood stasis (血瘀質)	4.23	6	10.85	14	6.42	7	10.81	12

**(B)** Percentage of participants reaching “Gentleness” BC after CM treatment				
	Baseline (V1)	After CM treatment (V4)	After CM treatment (V7)	Follow-up (V8)
Disease	80.28%	76.74%	72.48%	74.77%
Sub-Healthy (“Marginal Gentleness” BC)	11.27%	8.53%	11.93%	12.61%
Balanced (“Gentleness” BC)	8.45%	14.73%	15.60%	12.61%

**Fig. 1:**
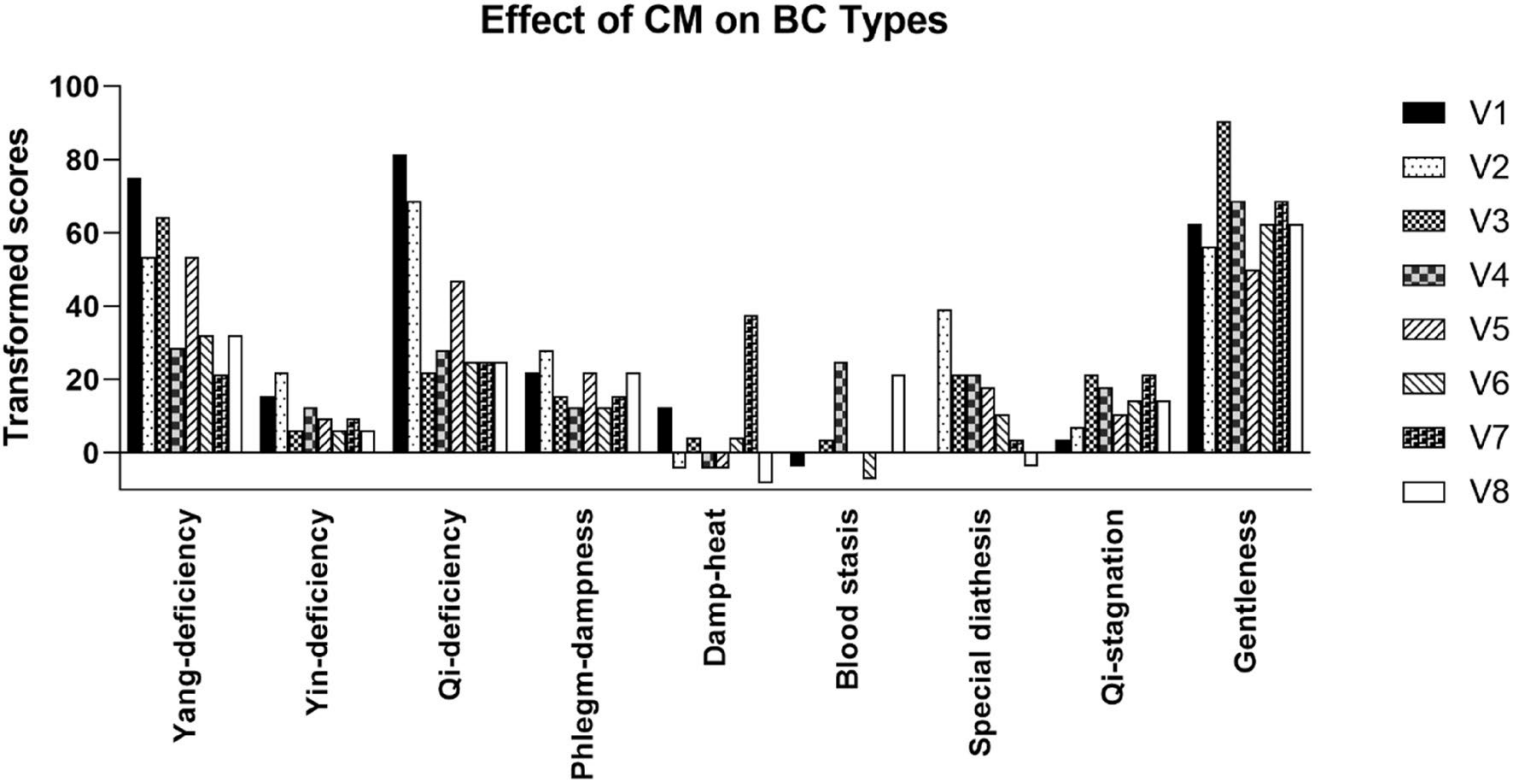
Effect of CM on BCs. Histogram showing the changes of each BC after CM treatment. Results are reported in BC transformed scores for each type of BC at different time points

On a summarized view of the change in BCs, it was found that the percentage of participants with unhealthy BCs decreased from 80 to 72% (Table 5B). Participants with a healthy BC status increased from 8.5% at baseline to 15.6% upon post-treatment. Considering a participant may exhibit a mixture of body constitutions, the actual effect of CM on each type of body constitution was further calculated using a transformed score for analysis. As shown in Fig. 1, it was found that scores of most unhealthy BCs had decreased after treatment (except Qi-stagnation). The Gentleness BC was observed to peak by V3.

**Fig. 2:**
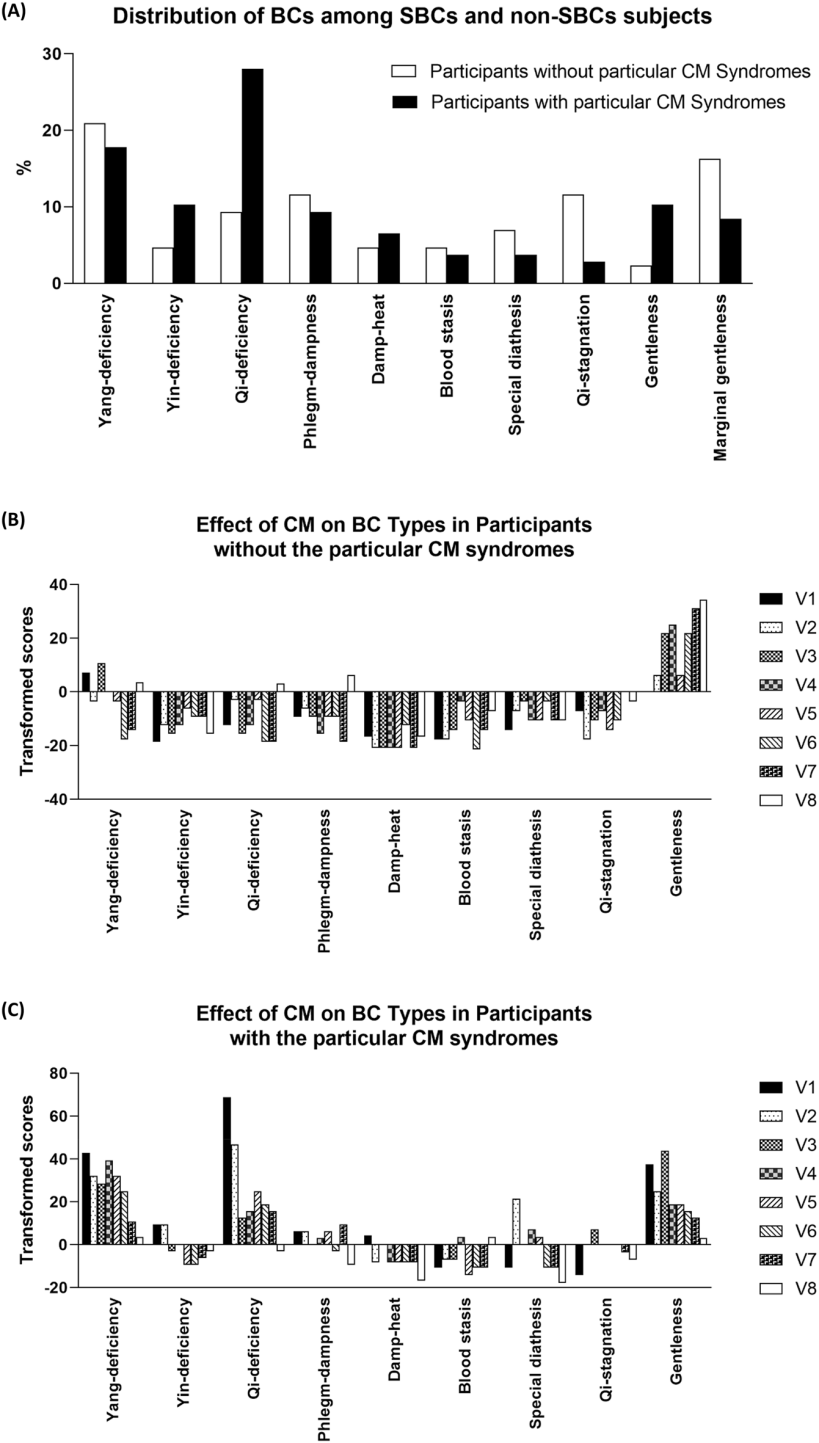
Histograms showing the **A** percentage of participants with or without the two particular CM Syndromes exhibiting each BC types, and the transformed scores of each BC assessed at different time point in participants (**B**) without the two particular CM Syndromes and (**C**) with the two particular CM Syndromes

The distribution of BCs among participants with or without the two particular CM Syndromes was also analyzed and distinct patterns were differentiated in the two groups of participants (Fig. 2).

Interestingly, it was found that participants without the particular CM Syndromes showed continue improvement in the healthy BC through V4 to V7 compared to those with the CM Syndromes that improvement was only observed up to V4 (Table 6). To investigate the recovery progress of the participants with the two particular CM Syndromes, the occurrence of clinical symptoms in these participants were analysed (Table 7). All main symptoms decreased after CM treatment and were found further diminished at follow-up assessment (Table 7A). For the accompanied clinical symptoms, a majority had decreased by the time of follow-up assessment (Table 7B).

**Table 6:** Changes in healthy (balanced), sub-healthy, and disease (unbalanced) BCs in participants within corresponding subgroups upon CM treatment and at follow-up

(A) Participants with particular CM syndromes				
% among group	Baseline (V1)	After CM treatment (V4)	After CM treatment (V7)	Follow-up (V8)
Disease	80.77	82.93	81.54	82
Sub-healthy	8.65	3.66	7.69	8
Healthy	10.58	13.41	10.77	10

(B) Participants without particular CM syndromes				
% among group	Baseline (V1)	After CM treatment (V4)	After CM treatment (V7)	Follow-up (V8)
Disease	78.95	65.96	59.09	68.85
Sub-healthy	18.42	17.02	18.18	16.39
Healthy	2.63	17.02	22.73	14.75

**Table 7:** CM effect on the occurrence (N, %) of clinical symptoms in participants diagnosed with one or both of the two particular CM Syndromes

(A) Main symptoms (主症)								
	Qi Deficiency of Lung and Spleen				Qi and Yin Deficiency			
	N	Cough	Coughing up phlegm	Shortness of breath	N	Cough	Coughing up phlegm	Shortness of breath
V1	92	43	32	42	80	34	29	36
		46.74%	34.78%	45.65%		42.50%	36.25%	45.00%
V2	85	37	27	39	77	30	24	35
		43.53%	31.76%	45.88%		38.96%	31.17%	45.45%
V3	79	35	25	36	64	30	21	27
		44.30%	31.65%	45.57%		46.88%	32.81%	42.19%
V4	72	26	26	31	65	30	22	22
		36.11%	36.11%	43.06%		46.15%	33.85%	33.85%
V5	66	22	23	26	64	21	21	22
		33.33%	34.85%	39.39%		32.81%	32.81%	34.38%
V6	59	21	24	23	56	21	21	25
		35.59%	40.68%	38.98%		37.50%	37.50%	44.64%
V7	58	18	23	20	49	14	20	20
		31.03%	39.66%	34.48%		28.57%	40.82%	40.82%
V8	39	12	9	16	35	8	10	11
		30.77%	23.08%	41.03%		22.86%	28.57%	31.43%

	(B) Accompanied symptoms (兼症)					
	Qi Deficiency of Lung and Spleen					
	N	Poor appetite	Fatigue	Spontaneous sweating	Post-meal fullness	Loose stool
V1	92	27	70	54	43	32
		29.35%	76.09%	58.70%	46.74%	34.78%
V2	85	19	62	50	33	20
		22.35%	72.94%	58.82%	38.82%	23.53%
V3	79	19	52	38	37	28
		24.05%	65.82%	48.10%	46.84%	35.44%
V4	72	24	47	36	30	17
		33.33%	65.28%	50.00%	41.67%	23.61%
V5	66	23	42	30	22	21
		34.85%	63.64%	45.45%	33.33%	31.82%
V6	59	19	38	32	21	14
		32.20%	64.41%	54.24%	35.59%	23.73%
V7	58	21	34	29	25	17
		36.21%	58.62%	50.00%	43.10%	29.31%
V8	39	12	25	20	17	9
		30.77%	64.10%	51.28%	43.59%	23.08%

	Qi and Yin Deficiency					
	N	Fatigue	Soreness and weakness of waist and knees	Feverish palms and soles	Tinnitus	Night sweats
V1	80	58	49	25	23	32
		72.50%	61.25%	31.25%	28.75%	40.00%
V2	77	53	56	19	20	16
		68.83%	72.73%	24.68%	25.97%	20.78%
V3	64	47	45	19	23	16
		73.44%	70.31%	29.69%	35.94%	25.00%
V4	65	44	49	24	22	18
		67.69%	75.38%	36.92%	33.85%	27.69%
V5	64	41	46	20	19	14
		64.06%	71.88%	31.25%	29.69%	21.88%
V6	56	38	38	16	16	13
		67.86%	67.86%	28.57%	28.57%	23.21%
V7	49	30	38	13	15	12
		61.22%	77.55%	26.53%	30.61%	24.49%
V8	35	19	23	8	6	10
		54.29%	65.71%	22.86%	17.14%	28.57%

### Improvement in 6MWT performance

**Fig. 3:**
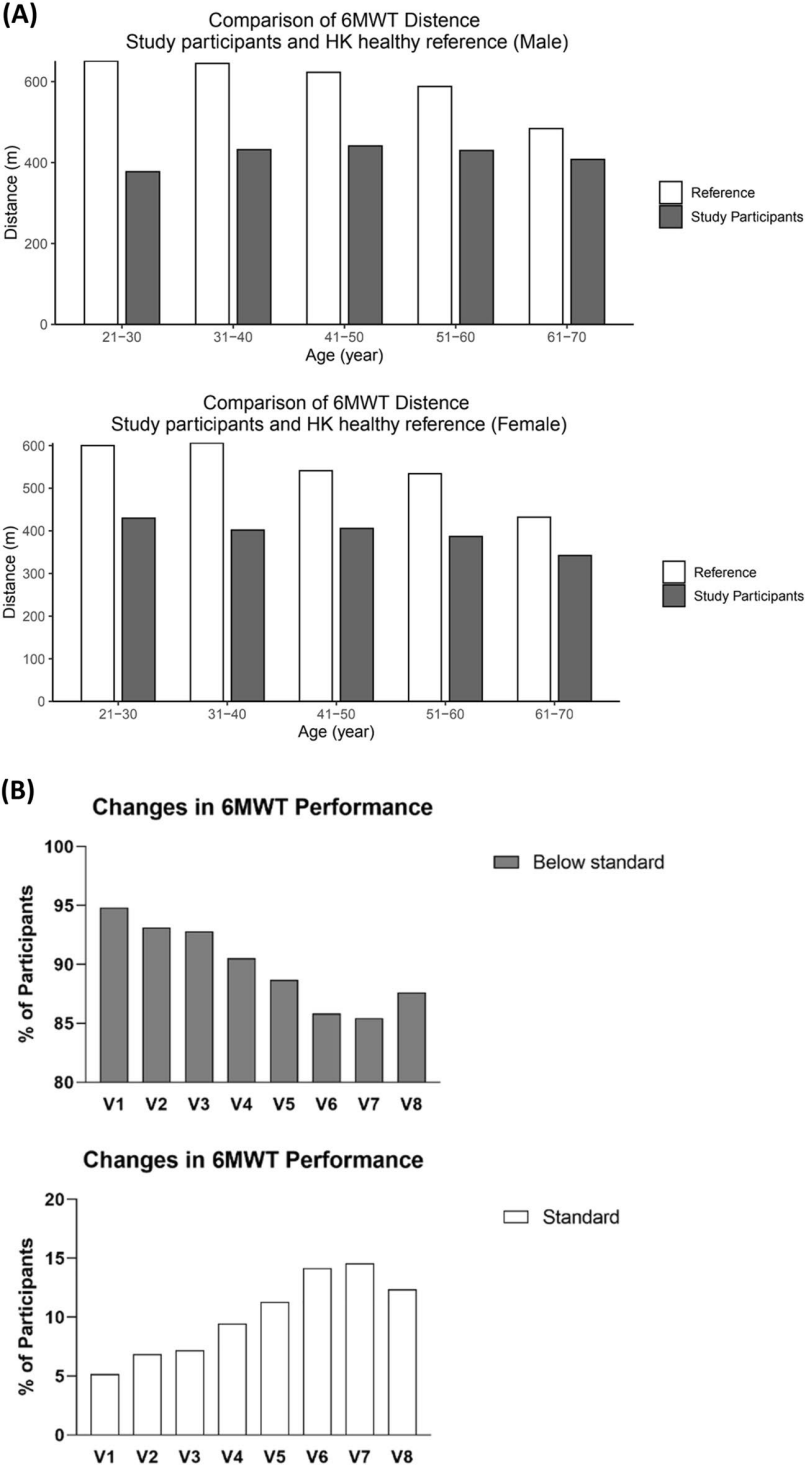
**A** Figure showed the participants’ baseline 6-MWT performance compared to the mean performance of the general HK population. **B** Figure showed the changes in percentage of participants achieving the general performance standards

Comparing to the corresponding age groups in the general HK population [38], the study participants showed a collectively lower performance in 6MWT across different age groups, irrespective of gender, at baseline assessment (Fig. 3A). For ages 21–70, male participants on average showed 57.97% of the general population performance in 6MWT while that for female participants was 71.61%. It was observed that 5.41% of the participants could achieve the general performance of the HK population while a 94.59% were below the general performance at the baseline assessment (V1) (Fig. 3B). The number of participants reaching the general performance of the population was found to increase from 5.41 to 9.56% at V4 (*P* = 0.365) and further to 13.91% at V7 (*P* = 0.031) as CM treatments were provided, however in the absence of treatments in the follow-up period, the percentage dropped to 11.97% (*P* = 0.268) at the follow-up period (V8). On the other hand, the number of participants who could not reach the general performance level decreased across time from 94.59 to 90.44% at V4 and further to 86.09% at V7 which returned to 88.03% at the follow-up period (V8).

**Fig. 4:**
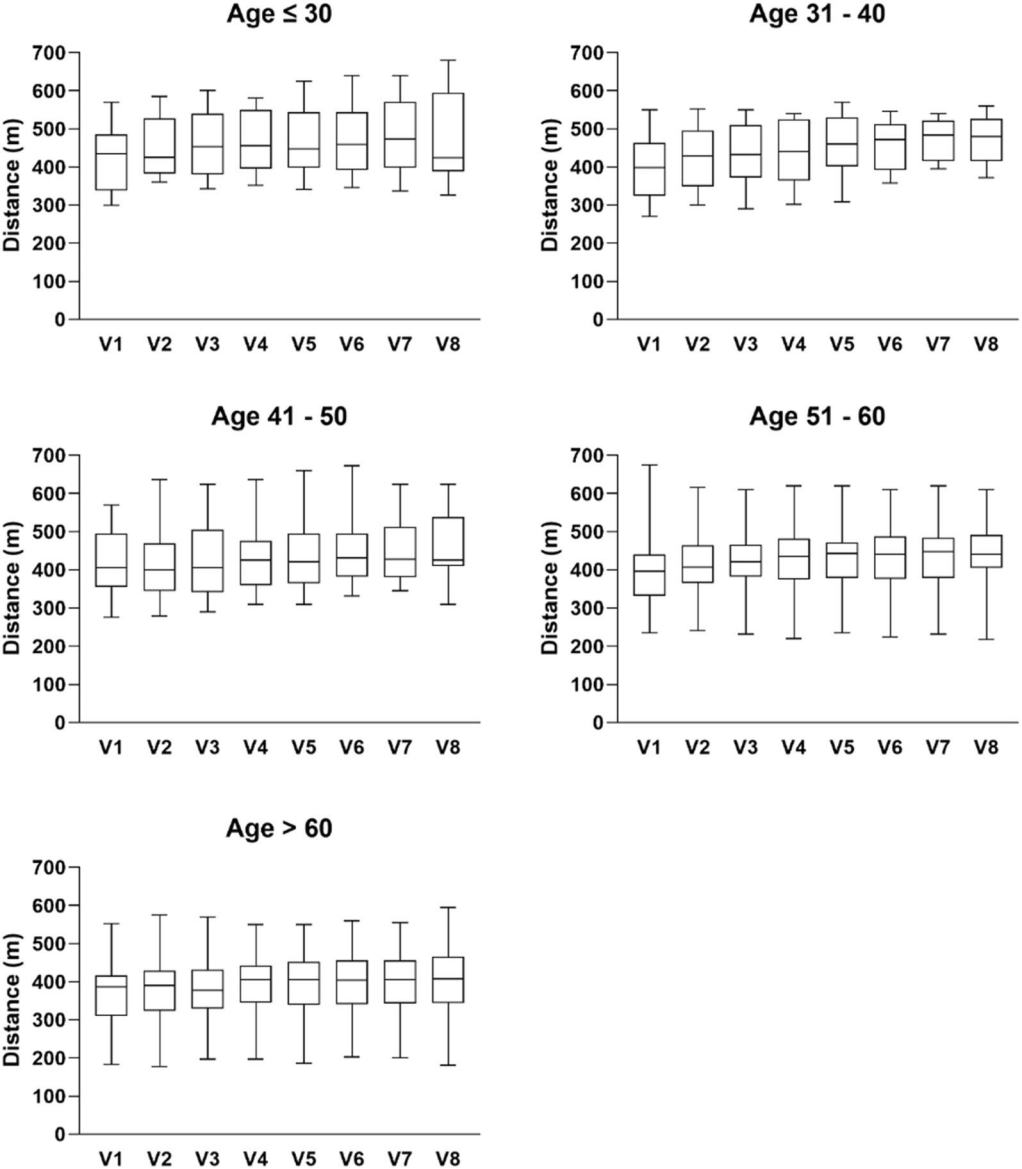
Distance achieved in 6MWT in different age groups

**Fig. 5:**
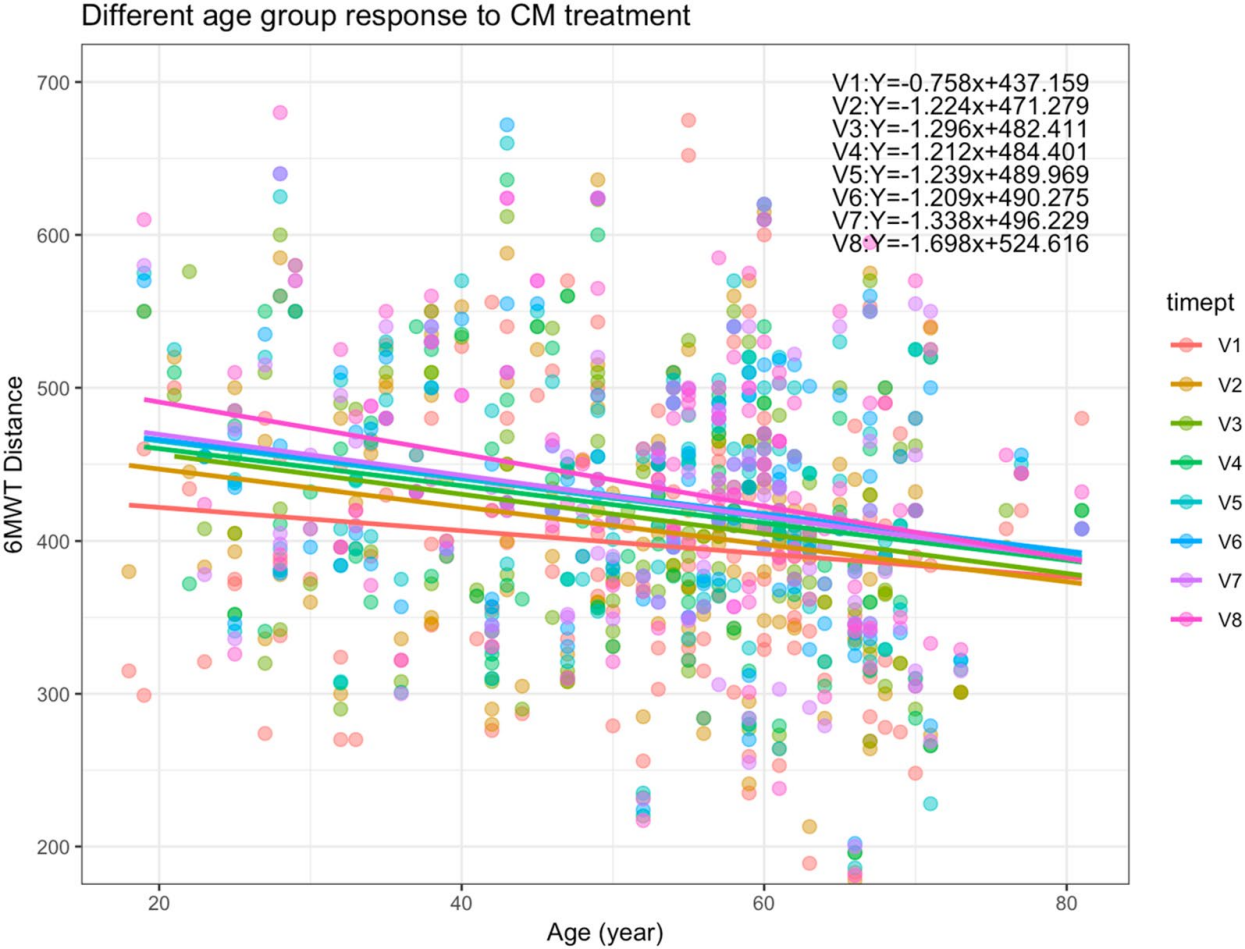
Performance of 6-MWT by participants receiving CM treatments over time and at follow-up period

To obtain a clear view of whether the response to CM treatment was affected by age, analysis was performed according to different age groups (Fig. 4). Considering there were two sub-groups of participants, the number of participants who could reach their predicted 6MWT distance was compared between the groups (Table 8). For an overview on the difference in 6MWT performance among participants of different age groups, Fig. 5 showed the linear regression lines for each CM treatment time points across different ages. There were observable differences in the slopes of each regression line.

**Table 8:** Frequency and percentage of group reaching predicted 6MWT distance

	Participants with particular CM syndromes			Participants without particular CM syndromes		
	N	Reached Predicted	Below Predicted	N	Reached Predicted	Below Predicted
V1	106	6	100	42	2	40
		5.66%	94.34%		4.76%	95.24%
V2	95	8	87	47	0	47
		8.42%	91.58%		0%	100%
V3	90	7	83	43	1	43
		7.78%	92.22%		2.27%	97.73%
V4	89	11	78	45	2	45
		12.36%	87.64%		4.26%	95.74%
V5	80	12	68	42	2	42
		15%	85%		4.55%	95.45%
V6	73	12	61	39	4	39
		16.44%	83.56%		9.3%	90.7%
V7	71	13	58	41	3	41
		18.31%	81.69%		6.82%	93.18%
V8	59	10	49	54	4	54
		16.95%	83.05%		6.9%	93.1%

Linear mixed effects analysis was conducted to elucidate the relationship between 6MWT performance and effect of CM treatment (Model 1). It was found that CM treatment had a highly significant effect on the distance achieved by participants in the 6MWT (Table 9A). Age was also found to significantly affect the 6MWT performance. It was noted that prior CM consultations or CM symptoms was close to affecting the 6MWT performance though not statistically significant. As shown in Table 9B, 6MWT performance was enhanced by increasing CM treatments. However, with each increased age year, the distance achieve in 6MWT will decrease by 0.961 m.

**Table 9:** Effect of CM treatment and age on the performance of participants in 6MWT

(A) Type III Analysis of Variance Table with Satterthwaite’s method of approximation for Model 1 of 6MWT distance			
Effect	Degrees of freedom (numerator, denominator)	F-value	p-value
CM visits	(879.83)	6.9087	< 0.001***
Age	(142.86)	4.0511	0.046*
CM symptoms	1010.59	0.2434	0.866
Prior CM consultations	(140.30)	3.0396	0.083

(B) Summary of fixed effects in Model 1			
Parameter	Estimate	Test (df)	Pr( >|t|)
(Intercept)	430.8429	t = 15.445 (148.4691)	< 0.001 ***
V2	10.0296	t = 2.289 (876.4085)	0.022 *
V3	15.7312	t = 3.514 (878.5520)	< 0.001***
V4	20.4666	t = 4.586 (879.3714)	< 0.001***
V5	22.5824	t = 4.924 (879.7408)	< 0.001***
V6	23.7805	t = 5.069 (880.7936)	< 0.001***
V7	21.0785	t = 4.475 (881.7175)	< 0.001***
V8	26.5497	t = 5.509 (895.2496)	< 0.001***
Age	− 0.9610	t = − 2.013 (142.8619)	0.046 *
Qi Deficiency of Lung and Spleen Syndrome only	2.2650	t = 0.290 (1009.1206)	0.772
Qi and Yin Deficiency only	− 6.1087	t = − 0.602 (1011.0674)	0.547
Both CM Syndromes	− 1.5165	t = − 0.236 (1021.3499)	0.814
Prior CM consultations	3.7064	t = 1.743 (140.2964)	0.083

Data were generated with a mixed-effects linear regression model, with fixed effects of CM treatment visits, age, CM symptoms, and prior CM consultations, while subjects as random effects (Model 1)

### Lowered risk of COPD

**Fig. 6:**
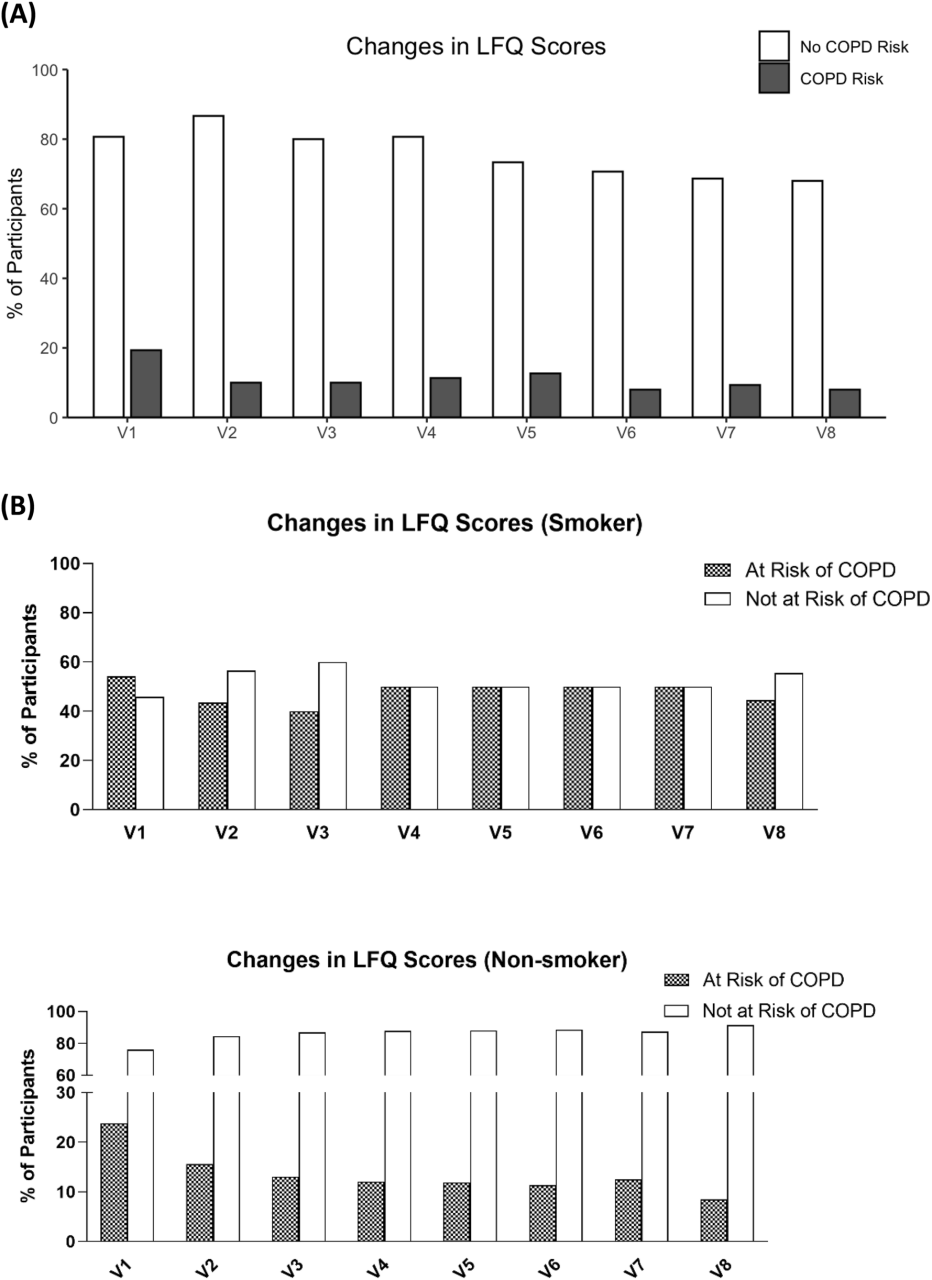
**A** Figure showed the percentage of participants with risk of COPD as accessed by their LFQ Scores. **B** Showed the percentage of non-smokers and smokers participants with risk of COPD

The impairment in lung function was also assessed in terms of the risk to the development of COPD. A score of below 18 in the LFQ indicated a risk of COPD in the participant. At baseline assessment, scores achieved by participants ranged from 13 to 25, with a median score of 20. As shown in Fig. 6A, the percentage of participants who would be risk free from COPD increased from 80.67 to 87.68% at V4 (*P* = 0.017) and further to 88.03% at V7 (*P* < 0.001) and then further to 89.47% (*P* < 0.001) at follow-up (V8). Those who would be at risk of COPD decreased from 19.33 to 11.97% at V4 and eventually to 10.53% at follow-up (V8).

**Fig. 7:**
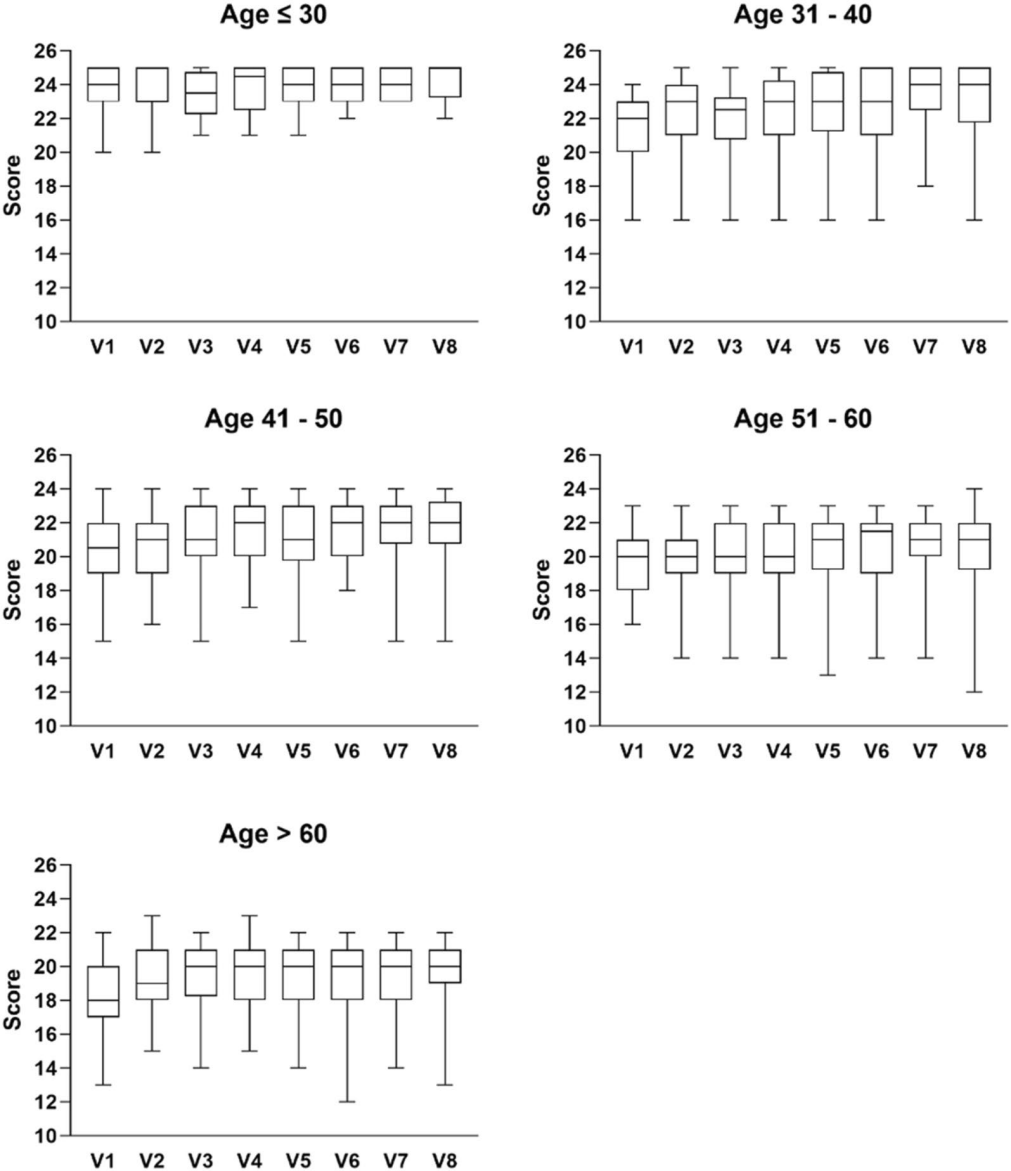
Scores achieved in LFQ in different age groups

Since smoking was considered to be a risk factor for development of COPD, analysis was conducted for smokers and non-smokers. As shown in Fig. 6B, it was observed that non-smokers had better improvements in decreasing the risk of COPD upon CM treatment compared to those of smokers. As a baseline difference was detected in LFQ scores between participants with the two particular CM Syndromes and those without, subgroup analysis was conducted. As shown in Table 10, COPD risks were shown to be decreased in both groups of participants. Although there were fewer smokers at baseline among the participants without the two CM Syndromes (18 smokers among those with the CM Syndromes and 6 among those without), the number of smokers increased in the group without the CM Syndromes as the smokers with the CM Syndromes did not exhibit the Syndromes anymore. Similar to the performance in 6MWT, LFQ scores were also analysed according to different age groups (Fig. 7). General observation showed an increase in median of LFQ scores upon treatments.

**Table 10:** Frequency and percentage of group with COPD Risk as assessed by LFQ Scores

	Participants with particular CM syndromes			Participants without particular CM syndromes		
	N	At risk	No risk	N	At risk	No risk
V1	107	24	84	42	5	37
		22.22%	77.78%		11.9%	88.1%
V2	99	11	88	46	4	42
		11.11%	88.89%		8.7%	91.3%
V3	86	14	78	43	1	42
		15.22%	84.78%		2.33%	97.67%
V4	86	13	78	47	4	43
		14.29%	85.71%		8.51%	91.49%
V5	81	15	68	46	4	42
		18.07%	81.93%		8.7%	91.3%
V6	72	9	66	43	3	40
		12%	88%		6.98%	93.02%
V7	67	9	64	44	5	39
		12.33%	87.67%		11.36%	88.64%
V8	50	6	53	55	6	49
		10.17%	89.83%		10.91%	89.09%

**Fig. 8:**
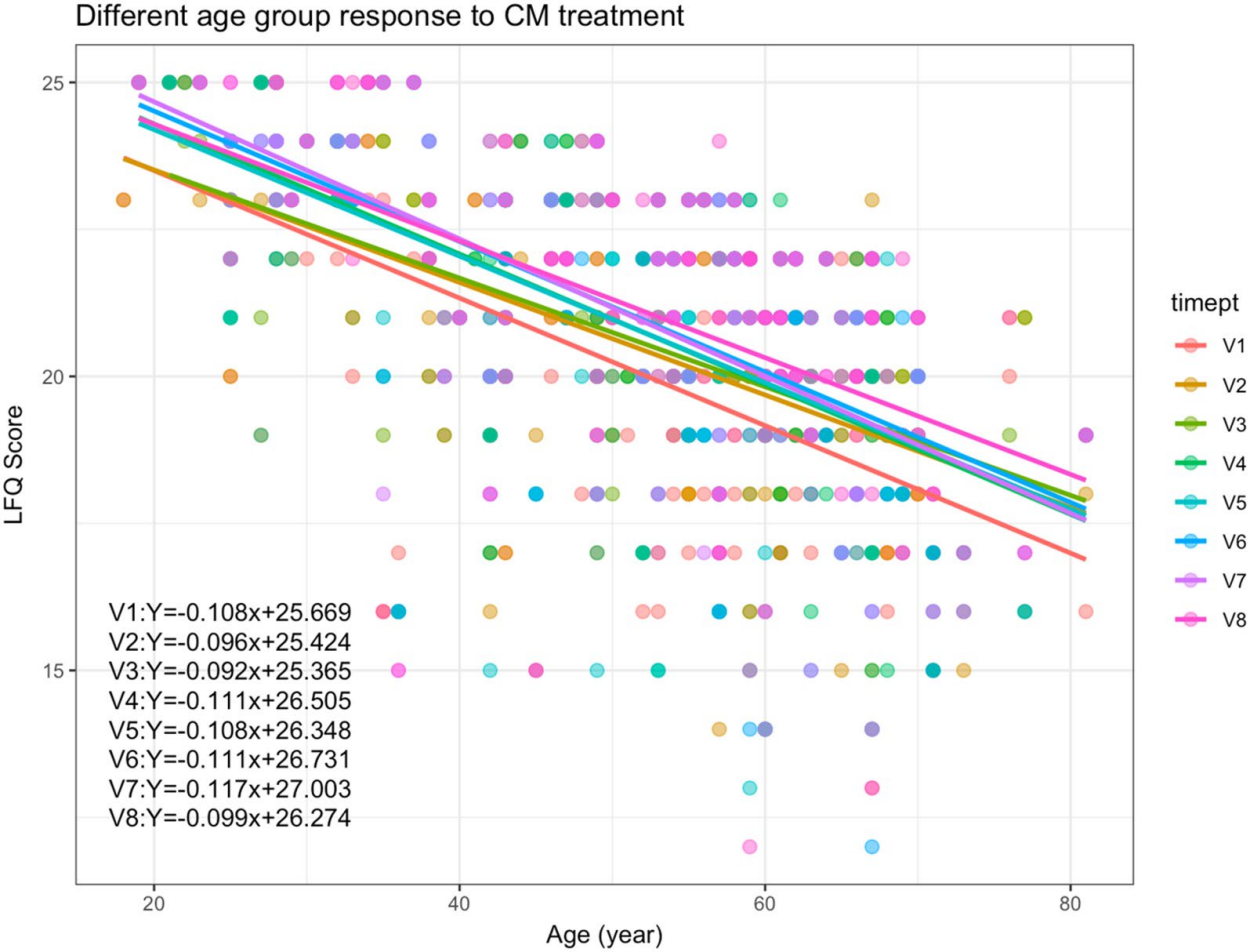
Changes in LFQ Scores of participants receiving CM treatments over time (V1–V7) and at follow-up (V8) period

For comprehensive view on the difference in LFQ score among participants of different age groups, Fig. 8 showed the linear regression lines for each CM treatment time points across different ages. There were observable differences in the slopes of each regression line.

Linear mixed effects analysis was conducted to elucidate the relationship between LFQ score and effect of CM treatment (Model 2). It was found that CM treatment had a highly significant effect on the LFQ score obtained by participants (Table 11A). It was noted that CM Syndromes showed little effect on the LFQ score obtained. As shown in Table 11B, LFQ score was enhanced by increasing CM treatments.

**Table 11:** Effect of CM treatment on the scores of participants in LFQ

(A) Type III Analysis of Variance Table with Satterthwaite’s method of approximation for Model 2 of LFQ score			
Effect	Degrees of freedom (numerator, denominator)	F-value	p-value
CM visits	(890.11)	14.774	< 0.001***
CM symptoms	(1, 022.48)	0.459	0.711

(B) Summary of fixed effects in Model 2			
Parameter	Estimate	Test (df)	Pr( >|t|)
(Intercept)	20.00396	t = 83.954 (290.58681)	< 0.001***
V2	0.46474	t = 3.960 (887.42330)	< 0.001***
V3	0.54977	t = 4.566 (889.33777)	< 0.001***
V4	0.77698	t = 6.490 (889.85982)	< 0.001***
V5	0.75384	t = 6.157 (890.29928)	< 0.001***
V6	0.95029	t = 7.538 (891.15305)	< 0.001***
V7	0.90848	t = 7.174 (892.23608)	< 0.001***
V8	1.10625	t = 8.457 (903.22758)	< 0.001***
Qi Deficiency of Lung and Spleen Syndrome only	− 0.04268	t = − 0.199 (1017.70401)	0.843
Qi and Yin Deficiency only	0.07637	t = 0.276 (1012.56804)	0.783
Both CM Syndromes	0.17216	t = 0.962 (1034.97834)	0.336

Data were generated with a mixed-effects linear regression model, with fixed effects of CM treatment visits and CM symptoms, while subjects as random effects (Model 2)

### Quality of Life

**Fig. 9:**
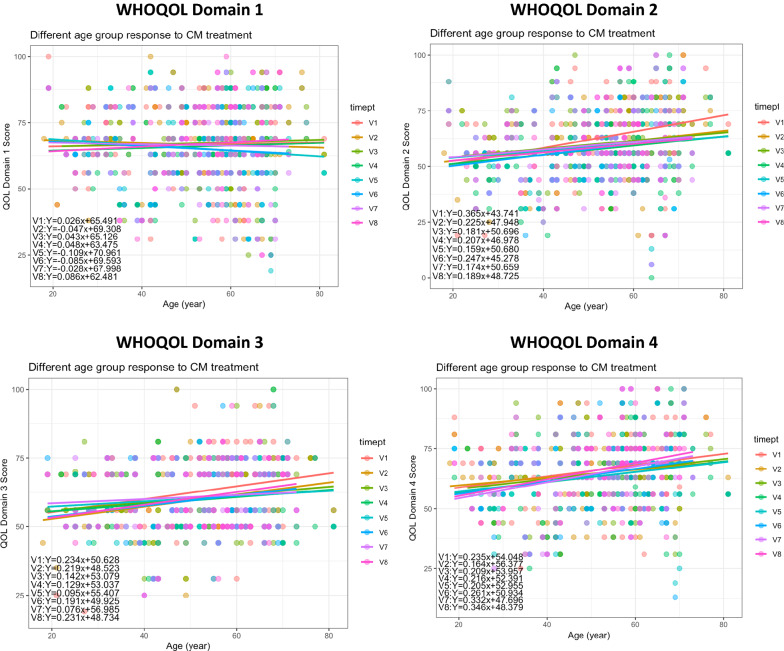
Changes in WHO-QOL domain scores of participants receiving CM treatments over time (V1–V7) and at follow-up (V8) period

By repeated measure t-test comparison between two CM treatment time points, it was found that quality of life in the aspects of psychological, social, and environment aspects were decreased upon CM treatment (Table 12A). Interestingly, further sub-group analysis regarding different age groups showed that CM treatment might have shown benefits for the participants of ages 31–40 (Table 12B). For an overview on the different QOL domain scores among participants of different ages, Fig. 9 showed the linear regression lines for each CM treatment time points across different ages. Due to a variety of confounding factors that were not yet taken into considerations in the above analysis, CM treatment did not show obvious trends for different ages.

**Table 12:** Changes in participants’ WHO-QoL Scores in different domains over the course of CM treatment

(A) General comparison of scores in different domains before and after CM treatment using t-test. Confounding factors were not taken into account in this brief analysis			
	V1	V4	P-value
D1	66.75	66.47580645	0.797389301
D2	62.78225806	58.35483871	5.94773E−05***
D3	62.4516129	59.80645161	0.007684351***
D4	66.24193548	63.51612903	0.006790345***

	V1	V7	P-value
D1	67.04210526	66.78947368	0.830492185
D2	63.16842105	60.33684211	0.0200676*
D3	63.69473684	61.05263158	0.026422436*
D4	67.12631579	65.26315789	0.113865638

(B) Overview of the percentage change in median domain scores (D1: Physical Health; D2: Psychological; D3: Social; D4: Environment) pre- and post-CM treatment for different age groups were shown. However, other confounding factors were not taken into account in this brief analysis				
Age group	D1	D2	D3	D4
Age < 21	− 6.4	− 8.0	10.4	− 18.3
Age 21–30	9.5	0.0	− 18.8	− 18.8
Age 31–40	4.3	6.3	11.6	16.0
Age 41–50	− 4.3	0.0	12.0	4.8
Age 51–60	0.0	− 13.8	− 18.8	− 8.7
Age 61–70	− 4.3	− 13.8	− 9.4	0.0
Age > 70	0.0	16.0	− 4.2	0.0

### Frequency of clinic/hospital visits

It was observed that most participants who received western medicine consultations during the study also had chronic disease history (Table 13), and almost all who required western medicine consultations related to flu or flu-like symptoms had chronic disease history. An observable decrease in participants requiring for non-flu related western medicine consultations was found during the CM treatment period.

**Table 13:** Number of participants receiving western medicine (WM) consultations during the study period

	No. of participants				No. of visits for WM consultations	
	Required WM consultations	With chronic disease history	Required WM consultations related to flu	With chronic disease history	Non-flu-related WM consultations	Flu-related WM consultations
V1	30	23	4	2	42	5
V2	20	14	3	2	25	4
V3	25	20	4	4	32	5
V4	22	18	4	4	25	4
V5	18	13	3	2	28	4
V6	19	15	2	2	24	2
V7	15	13	2	2	22	3
V8	27	21	3	2	38	4

## Discussion

This study is to our knowledge the first multicenter observational study in Hong Kong on post-COVID patients’ rehabilitation that reports from CM perspectives, with standardized CM assessments as well as quantitative assessments on lung functions. Current observations showed that CM could facilitate the resolution of several post-COVID clinical symptoms including cough, fatigue, and shortness of breath, and some gastrointestinal symptoms such as post-meal fullness and loose stool. Furthermore, results showed an improvement trend in the lung functions (in terms of performance in 6MWT and risk of COPD as assessed by LFQ) of participants receiving CM treatment. During treatment period, the number of participants achieving a balanced BC observably increased.

COVID-19 symptoms have been reported to persist after hospital discharge [39, 40]. Such symptoms not only included impairment in lung physiology and radiographic change [39] but also in physical and psychological aspects such as fatigue, sleep difficulties, and anxiety [40]. In our study, participants suffered persistent symptoms such as fatigue and muscle pain, cough with or without phlegm, shortness of breath, abnormal sweating, tinnitus, and gastrointestinal symptoms. Most symptoms were resolved after CM treatment though not all participants showed the same degree of responses to CM treatment. This could be due to the sub-groups of participants with different CM Syndrome types. On sub-group analysis of those with particular CM Syndromes, however, there were no difference in their lung function improvements.

In addition to clinical symptoms, this study found that participants’ BC improved, with more participants achieved a balanced (“Gentleness”) BC after CM treatment. Interestingly, it was found in sub-group analysis that participants without the two particular CM Syndromes showed better response to CM treatment on achieving balanced BC. Although the concept of body constitution was conventionally applied in CM [41], only in recent years there emerged research publications in English scientific journals, providing evidence on the association between different types of BCs with disease or abnormal health conditions, linking a CM assessment method with western medicine disease classifications [42–47].

In this study, there were participants who did not exhibit the two particular CM Syndromes that were indicated in post-COVID patients. Interestingly, this sub-group of participants showed better recovery compared to those with the two CM Syndromes. This observation suggested a potential in exploring the application of CM Syndrome differentiation in adjusting different health management strategies for post-COVID patients with different types of post-COVID sequela. A recent study by Xie et al. showed that there is a substantial risk of cardiovascular disease in post-COVID patients [48]. Health problems have been reported after COVID-19 vaccination such as myocarditis and pericarditis [49, 50]. Apart from myocarditis, a variety of health problems have also been reported post-vaccination, such as deep-vein thrombosis, myocardial infarction, and thrombocytopenia [51]. It is worthwhile to further explore the usage of CM diagnosis and treatment in early prevention or treatment of these problems. Collaborative efforts of multi-disciplinary branch would be beneficial to patients’ recovery and rehabilitation [51–53]. The demand for western medicine consultations for post-COVID participants in this study had decreased when CM was provided. This observation suggested CM as a favorable alternative medical care for these patients in Hong Kong.

A cross-sectional study by Mandal et al. showed some spontaneous resolution of symptoms and restoration of lung functions in post-COVID subjects [54]. Data in current study found that both only the subgroup with the particular CM Syndromes showed improvement in 6MWT performance and better response in the assessments for COPD risks. Our observations may suggest the need to identify subjects who would benefit most with individualized CM care in post-discharge recovery.

In the current study, several limitations were identified. One of the most important limitations to be considered in this study was the relatively small sample size for further sub-group analysis. Another limitation to the current study was the lack of CM Syndrome assessments in COVID-19 patients upon their hospitalization. Identification of CM Syndromes upon hospitalization during acute COVID-19 phase would have allowed for analysing the transitions of Syndrome types, as well as BCs, in patients from admission to discharge to rehabilitation, and hence to elucidate appropriate individualized CM treatments based on their health change. Furthermore, the quality of life assessment in the current study might have been affected by a variety of confounding factors which were not recorded. Despite these limitations, the current findings suggested a positive role of CM in parallel to western medicine in facing the immense challenge posed by COVID-19.

An essential feature of CM is the holistic concept, in which treatment is based on syndrome differentiation instead of individual clinical symptom, and healthcare advice is provided to patients with consideration to their lifestyle and habits. The main principle of CM is to achieve balance (such as the balance of Yin and Yang in one’s health), hence strengthening body resistance to eliminate pathogenic factors as well as leading a healthy lifestyle. In light of the healthcare burden inflicted by the pandemic and subsequent post-COVID rehabilitation, CM would offer additional treatment options while at the same time bring in new opportunities for exploration in areas where CM could assist in diagnoses that are ambiguous from the western medicine perspective.

## Conclusion

This study provided evidence for individualized CM treatment on COVID-19 rehabilitation concerning the reduction in clinical symptoms, achieving balance in body constitution, and improvement of lung functions. It is believed that CM may be a key to further promote rehabilitation and resolution of residual symptoms. Long-term follow-up studies on persistent symptoms would be required to further examine CM effects on long-COVID symptoms.

## Supplementary Information

**Additional file 1**. Tables S1 and S2 and Appendix S1 and S2.

## Data Availability

All data sources described in this research are available from the corresponding authors.
