# Supplementary material for: Effects of Chinese medicine for COVID-19 rehabilitation: a multicenter observational study
Source: Chin Med. 2022 Aug 22;17:99. doi: 10.1186/s13020-022-00654-z (PMC9395813; doi:10.1186/s13020-022-00654-z)
Supplement: Supplementary file 1 — Additional file 1. Tables S1 and S2 and Appendix S1 and S2. [file 13020_2022_654_MOESM1_ESM.docx]

**Table S1.**

The recommended prescriptions for the two particular CM syndromes in COVID-19 rehabilitation and their corresponding clinical manifestations are shown below:

| **CM Syndrome** | **Clinical Manifestations** | **Recommended Prescription** |
| --- | --- | --- |
| Qi Deficiency of Lung and Spleen  (“肺脾兩虛”) | - shortness of breath - fatigue - anorexia - nausea - fullness - weak stool - uneasiness - pale and greasy tongue | - French Pinellia, 9g - Chenpi, 10g - Codonopsis, 15g - Sunburn Astragalus, 30g - Stir-fried Atractylodes, 10g - Poria, 15g - Huoxiang, 10g - Amomum villosum, 6g (later) - Licorice, 6g |
| Qi and Yin Deficiency  (“氣陰兩虛”) | - fatigue - shortness of breath - dry mouth, thirst - palpitations - sweating - poor appetite - low or no lever - dry cough and little sputum - dry tongue - fine or weak pulses | - North and south radix salvia, 10g, 15g - Ophiopogonis, 6g - American ginseng, 6g - Schisandra, 6g - Gypsum, l5g - Light bamboo leaves, 10g - Mulberry leaves, 10g - Reed root, 15g - Salviae miltiorrhiza, 15g - Raw liquorice, 6g |

**Table S2.** Schedule for Outcome Measurement

| **Assessment** | **Baseline** | **Program Intervention** | | | | | | **Follow-up** |
| --- | --- | --- | --- | --- | --- | --- | --- | --- |
| Visit | 1 | 2 | 3 | 4 | 5* | 6* | 7* | 8 |
| Study Months | 0 | 1 | 2 | 3 | 4* | 5* | 6* | 9 |
| Informed Consent | √ |  |  |  |  |  |  |  |
| History | √ |  |  |  |  |  |  |  |
| CM Diagnostic Pattern  & Clinical Characteristics Assessments | √ | √ | √ | √ | √ | √ | √ | √ |
| Lung Function Assessments  6MWT  LFQ  FEV/FVC (if required) | √ | √ | √ | √ | √ | √ | √ | √ |
| Quality of Life Assessments  (WHO-QOL BREF HK) | √ | √ | √ | √ | √ | √ | √ | √ |
| Body Constitution Assessment  CCMQ Questionnaire | √ | √ | √ | √ | √ | √ | √ | √ |
| Assessments on the Frequency of Western Medicine (WM) Consultations | √ | √ | √ | √ | √ | √ | √ | √ |

* The extended treatment is optional.

Remarks: There will be 8 study visits in total.

Visit 1: Baseline visit

Visit 2 to Visit 7: Patients will receive Chinese Medicine treatment and will be assessed by questionnaires and lung function tests on the same day.

Visit 8: follow-up visit after 3 months later

**Appendix S1.**

Government subsidized tri-partite CM Clinic cum Training and Research Centres that participated in this study are listed below:

1. The Hong Kong Tuberculosis Association - The University of Hong Kong Chinese Medicine Clinic cum Training and Research Centre (Southern District)
2. Haven of Hope - The Chinese University of Hong Kong Chinese Medicine Clinic cum Training and Research Centre (Sai Kung District)
3. Pok Oi Hospital - Hong Kong Baptist University Chinese Medicine Clinic cum Training and Research Centre (Kowloon City District)
4. Pok Oi Hospital- The Chinese University of Hong Kong Chinese Medicine Clinic cum Training and Research Centre (Shatin District)
5. Hong Kong Federation of Trade Unions Workers' Medical Clinics - Hong Kong Baptist University Chinese Medicine Clinic cum Training and Research Centre (North District)
6. United Christian Nethersole Community Health Service - The Chinese University of Hong Kong Chinese Medicine Clinic cum Training and Research Centre (Tai Po District)
7. Pok Oi Hospital - The Chinese University of Hong Kong Chinese Medicine Clinic cum Training and Research Centre (Yuen Long District)

**Appendix S2.**

**I．肺脾兩虛**

主症：

| 咳嗽 | 0（ ） 2（ ） 4（ ） 6（ ） |
| --- | --- |
| 咯痰 | 0（ ） 2（ ） 4（ ） 6（ ） |
| 氣喘 | 0（ ） 2（ ） 4（ ） 6（ ） |

兼症：

| 納差 | 0（ ） 1（ ） 2（ ） 3（ ） |
| --- | --- |
| 乏力 | 0（ ） 1（ ） 2（ ） 3（ ） |
| 自汗 | 0（ ） 1（ ） 2（ ） 3（ ） |
| 食後脘痞 | 0（ ） 1（ ） 2（ ） 3（ ） |
| 便溏 | 0（ ） 1（ ） 2（ ） 3（ ） |

評分標準：

| 主症 | 0 | 2 | 4 | 6 |
| --- | --- | --- | --- | --- |
| 咳嗽 | 無 | 偶咳 | 稍咳嗽或夜裡偶咳，不影響工作生活 | 晝夜頻咳，影響工作生活 |
| 咯痰 | 無 | 日咳痰 10-50mL | 日咳痰 50~100mL | 日咳痰 100mL以上 |
| 氣喘 | 無 | 重體力活動後發生 | 輕度活動後發生，不影響睡眠 | 無法平臥，影響睡眠及活動 |

| 兼症 | 0 | 1 | 2 | 3 |
| --- | --- | --- | --- | --- |
| 納差 | 無 | 食欲稍減，食量不變 | 食欲減退，食量減少 1/3 以內 | 無食欲，食量減少 1/3 以上 |
| 食後脘痞 | 無 | 食後半小時內緩解 | 食後 1/2 - 2h 內緩解 | 持續脘悶腹脹 |
| 自汗 | 無 | 輕度活動後偶有 | 輕度活動後常有 | 休息時有，動則明顯 |
| 乏力 | 無 | 稍有，不影響工作生活 | 有，尚可接受輕體力活 | 明顯，無法工作、自主生活 |
| 便溏 | 無 | 日 1 次 | 日 2 – 4 次 | 日 4 次以上 |

**II．氣陰兩虛**

主症：

| 咳嗽 | 0（ ） 2（ ） 4（ ） 6（ ） |
| --- | --- |
| 咯痰 | 0（ ） 2（ ） 4（ ） 6（ ） |
| 氣喘 | 0（ ） 2（ ） 4（ ） 6（ ） |

兼症：

| 乏力 | 0（ ） 1（ ） 2（ ） 3（ ） |
| --- | --- |
| 腰膝酸軟 | 0（ ） 1（ ） 2（ ） 3（ ） |
| 手足心熱 | 0（ ） 1（ ） 2（ ） 3（ ） |
| 耳鳴 | 0（ ） 1（ ） 2（ ） 3（ ） |
| 盜汗 | 0（ ） 1（ ） 2（ ） 3（ ） |

評分標準：

| 主症 | 0 | 2 | 4 | 6 |
| --- | --- | --- | --- | --- |
| 咳嗽 | 無 | 偶咳 | 經常咳嗽，但能自止 | 咳嗽頻繁，常不自止 |
| 咯痰 | 無痰 | 咳痰量少而稀 | 咳痰量少而黏 | 痰少不易出，咳吐不爽 |
| 氣喘 | 無 | 偶有，大量活動後加重 | 活動後加重，輕度影響日常活動 | 輕度活動後既有症狀，影響日常活動 |

| 兼症 | 0 | 1 | 2 | 3 |
| --- | --- | --- | --- | --- |
| 乏力 | 無 | 偶有乏力 | 經常乏力，不影響日常活動 | 乏力明顯，影響日常活動 |
| 腰膝酸軟 | 無 | 偶有腰膝酸軟，不影響日常活動 | 時有腰膝酸軟，活動後明顯，休息可緩解 | 總是腰膝酸軟，影響日常活動 |
| 手足心熱 | 無 | 偶有手足心熱 | 時有手足心熱，心中煩熱 | 總是手足心熱，甚心中煩悶，夜間影響入眠 |
| 耳鳴 | 無 | 偶有耳鳴，但不覺痛苦 | 持續性耳鳴，安靜時明顯 | 持續性耳鳴，影響日常生活作息 |
| 盜汗 | 無 | 偶有盜汗 | 時有盜汗，身有汗感 | 經常盜汗，汗濕衣被 |
